# Reliability and Validation of the Vietnamese Utian Quality-of-Life Scale in Postmenopausal Women

**DOI:** 10.3390/ijerph23060798

**Published:** 2026-06-15

**Authors:** Nguyen Dinh Phuong Thao, Le Thi Thanh Tuyen, Dao Trong Quan, Duong Thi Kim Hoa

**Affiliations:** 1 Faculty of Medicine, Da Nang University of Medical Technology and Pharmacy, 99 Hung Vuong Street, Hai Chau Ward, Da Nang 550000, Vietnam; ndpthao@dhktyduocdn.edu.vn (N.D.P.T.); dtkhoa@dhktyduocdn.edu.vn (D.T.K.H.); 2 School of Medicine and Pharmacy, The University of Da Nang, Ngu Hanh Son Ward, Da Nang 550000, Vietnam; 3 Faculty of Nursing, Thai Nguyen University of Medicine and Pharmacy, 284 Luong Ngoc Quyen Street, Phan Dinh Phung, Thai Nguyen 250000, Vietnam; daotrongquan@tnmc.edu.vn

**Keywords:** menopause, quality of life, Vietnamese women, UQOL, psychometric validation

## Abstract

**Highlights:**

**Public health relevance—How does this work relate to a public health issue?**

- Menopause affects multiple dimensions of women’s well-being, yet no culturally adapted instrument exists to assess menopause-specific quality of life in Vietnamese women, limiting an accurate evaluation of the health needs of this growing population.
- The Vietnamese Utian Quality of Life (VN-UQOL) Scale addresses this gap by providing a validated tool to measure occupational, health, emotional, and sexual quality of life in postmenopausal women, enabling a comprehensive assessment beyond symptom-based approaches.

**Public health significance—Why is this work of significance to public health?**

- The VN-UQOL demonstrates strong psychometric properties (Cronbach’s α = 0.87 overall; sub-scales 0.81–0.93), confirming its reliability and validity for use in community-based research and clinical practice with Vietnamese postmenopausal women.
- Successful cultural adaptation of the UQOL facilitates Vietnam’s participation in international menopause research and enables cross-country comparisons, contributing to the global understanding of menopause-related quality of life across diverse populations.

**Public health implications—What are the key implications or messages for practitioners, policymakers and/or researchers in public health?**

- Clinicians now have access to a brief, standardized, culturally appropriate instrument (23 items, 15–20 min) to identify women with compromised quality of life and monitoring responses to interventions in primary care, gynecology, and menopause specialty settings.
- Researchers can utilize the VN-UQOL as a validated outcome measure for studies examining menopause transitions, evaluating interventions, and informing health-service planning for the growing population of midlife women in Vietnam.

**Abstract:**

The absence of a culturally adapted instrument to assess menopause-specific quality of life in Vietnamese women limits both clinical practice and research in this population. This study aimed to translate, culturally adapt, and psychometrically validate the Vietnamese version of the Utian Quality of Life (VN-UQOL) Scale. A cross-sectional design was employed with 384 community-dwelling postmenopausal women aged 46–65 years. The UQOL was translated and adapted following established guidelines, including forward-back translation, expert review, and pilot testing. Internal consistency was evaluated using Cronbach’s alpha, construct validity through confirmatory factor analysis (CFA), and concurrent validity via Pearson correlations with the Vietnamese Menopause Rating Scale (VN-MRS). The VN-UQOL demonstrated excellent internal consistency, with a total scale Cronbach’s alpha of 0.87 and sub-scale alphas ranging from 0.81 to 0.93. Content validity indices (I-CVI, S-CVI) were 1.0. CFA confirmed the original four-factor structure, with all factor loadings exceeding 0.50 and good model fit indices (CFI = 0.921, RMSEA = 0.072). Concurrent validity was supported by significant negative correlations between the VN-UQOL sub-scales and corresponding VN-MRS domains (*p* < 0.01). The VN-UQOL is a reliable and valid instrument for assessing menopause-specific quality of life in Vietnamese women, providing a valuable tool for clinical practice and research in this underserved population.

## 1. Introduction

Menopause is a universal midlife transition with substantial implications for women’s quality of life (QOL). Beyond vasomotor and urogenital symptoms, menopause affects psychological well-being, family and social roles, occupational functioning, and sexual health, making QOL a central outcome in both clinical practice and research on midlife women. Conventional menopause instruments have often focused narrowly on symptom counts or disease-oriented indices, rather than on women’s broader sense of well-being across life domains [1]. This conceptual limitation has driven the development of more comprehensive, menopause-specific QOL measures.

The Utian Quality of Life (UQOL) Scale was introduced to address this gap by operationalizing QOL as perceived well-being in four domains: occupational, health, emotional, and sexual quality of life. In its original validation among 597 peri- and postmenopausal women in 12 U.S. communities (mean age 52.9 years), factor analysis supported this four-factor structure and yielded a 23-item instrument with good psychometric properties. Test–retest correlations for the four domains ranged from 0.752 to 0.887, and Cronbach’s α coefficients ranged from 0.656 to 0.876, with an overall α of 0.830, indicating acceptable stability and internal consistency for both total and sub-scale scores. Convergent validity with the widely used SF-36 further supported its use as a robust outcome measure in menopause research [1]. However, the original validation and subsequent replications have almost focused to confirm a four-factor structure, and may raise the possibility that a more general, overarching quality-of-life factor underlies the domain-specific factors. The current study therefore would not only test the original four-factor model but also examine competing structures.

Given marked cross-cultural variation in the meaning of menopause, symptom expression, and role expectations, culturally adapted instruments are essential for valid assessment. Evidence from Turkey demonstrates that relying on generic or non-adapted tools can underestimate culturally specific concerns. There, the lack of menopause-specific scales prompted translation and validation of the Turkish UQOL (UQOL-T), which proved to be psychometrically robust and practical for routine use [2]. Similarly, in Nigeria, the Yoruba UQOL (YUQOL) was needed because English-language instruments could not adequately capture the QOL in an indigenous African language context [3]. Moreover, even after careful adaptation, sub-scales showed weak convergent validity, such as the sexual sub-scale of the YUQOL [3]. This pattern suggests that in settings with conservative sexual norms like Vietnam, unadapted or poorly adapted instruments may seriously misrepresent women’s sexual and relational well-being during the menopausal transition.

Vietnam is undergoing rapid demographic aging, with a growing population of postmenopausal women who will spend a substantial proportion of life beyond reproductive age. In this context, menopause-related morbidity, including vasomotor symptoms, psychological distress, and sexual dysfunction, interacts with a rising burden of non-communicable diseases such as osteoporosis, cardiovascular disease, and diabetes, making QOL a critical patient-centered outcome. However, no validated Vietnamese menopause-specific QOL instrument currently exists. This gap has direct clinical and epidemiological implications, as without a standardized tool, clinicians in primary care and women’s health settings cannot systematically identify women with impaired QOL, monitor treatment response, or compare outcomes across facilities. From a public health perspective, the absence of a validated instrument limits Vietnam’s capacity to generate internationally comparable data, conduct multicenter research, and evaluate the effectiveness of health policies and interventions targeting midlife women’s well-being. Thus, this study aims to translate, culturally adapt, and psychometrically validate the UQOL for Vietnamese postmenopausal women. A rigorously developed Vietnamese UQOL would fill a critical methodological gap, enabling culturally sensitive assessment, informing health-service planning for midlife women, and providing a standardized outcome measure for clinical and public-health interventions targeting the menopausal transition in Vietnam.

## 2. Materials and Methods

Study design: Cross-sectional study design.

Sample: The study population comprised women who had undergone natural menopause, defined as the absence of menstruation for at least 12 consecutive months without resumption.

Inclusion Criteria: Women were eligible for inclusion if they met the following criteria:

1. Under 65 years old (following the WHO definition as the beginning of old age);
2. Confirmed natural menopause with no return of menstruation after 12 consecutive months;
3. No current or prior use of any form of hormone therapy.

Exclusion Criteria: Participants were excluded if they met any of the following conditions:

1. Diagnosis of malignancy or psychiatric disorders;
2. History of total hysterectomy with bilateral salpingo-oophorectomy performed either before or after menopause.
3. Inability to communicate effectively or cognitive impairment that precluded accurate responses to the interview questions.

### 2.1. Sample Size

To ensure construct validity and reliability, data should be collected from a sufficiently large and appropriately representative sample of the target population. A commonly accepted rule of thumb recommends a minimum of 10 participants per item of the scale, with an ideal participant-to-item ratio of 15:1 or 20:1 [4,5,6]. In the present study, a ratio of 15:1 was adopted. Given that the instrument comprised 23 items, the minimum required sample size was calculated as *n* = 23 × 15 = 345. Ultimately, a total of 384 participants were recruited for the study, exceeding the minimum requirement.

### 2.2. Sampling Technique

A list of wards and the number of menopausal women in each ward within Da Nang city was compiled. The sample size for each ward was selected proportionally to the number of menopausal women in that ward, employing a multi-stage cluster sampling technique. In stage 1, the study communes/wards were selected: 30% of the wards/communes, corresponding to 17 wards/communes, were randomly selected for inclusion in the study using simple random sampling. In stage 2, the study villages/hamlets were selected: 30% of the villages/hamlets within each selected commune/ward were randomly chosen for the study area. In stage 3, the study subjects were selected. A sampling frame was established, consisting of all eligible menopausal women residing in the selected villages/hamlets. Subjects were selected using systematic random sampling. A random number smaller than the sampling interval *k* (*k* ≤ 5) was chosen using a random number table; subsequently, 1 in every 5 menopausal women from that ward was selected. For example, if the first selected subject was at serial number 3, the next selected subject would be at serial number 8.

### 2.3. Instrument

The study used three questionnaires as follows:

The demographic questionnaire comprised two questions about age and education level.

Utian Quality of Life Scale (UQOL): UQOL was developed by Utian and colleagues in 2002. The UQOL is a 23-item instrument comprising four distinct domains: occupational quality of life (items 2, 3, 6, 17, 18, 19, and 23); health-related quality of life (items 7, 8, 9, 10, 16, 21, and 22); emotional quality of life (items 1, 11, 12, 13, 15, and 20), and sexual quality of life (items 4, 5, and 14). Responses to each item were recorded using a five-point Likert-type scale, where 1 indicated “not true of me” and 5 indicated “very true of me”. Total obtainable scores on the questionnaire range from 23 to 115, with higher scores on both the overall scale and its subdomains reflecting a more favorable quality of life. In the original validation study, the UQOL demonstrated robust internal consistency, with a Cronbach’s alpha coefficient of 0.83 [1]. Permission to utilize the instrument and adapt it into the Vietnamese language was formally obtained from Professor Utian.

Back-translation process

The back-translation process employed in this study adhered to the systematic guidelines proposed by Sousa and Rojjanasrirat to ensure semantic, conceptual, and content equivalence between the original English instruments and their Vietnamese adaptations. The multi-step procedure commenced with two independent forward translations by bilingual translators with distinct professional backgrounds—one in obstetrics and one in English language instruction—to capture both technical accuracy and colloquial appropriateness. These versions were subsequently synthesized by a third independent translator and a review committee. This preliminary Vietnamese version then underwent blind back-translation by two additional bilingual translators, whose outputs were compared against the original instrument to identify and rectify discrepancies in meaning, grammar, and cultural relevance through multidisciplinary committee consensus [7].

The Vietnamese version of the UQL (VN-QOL) then was tested with 40 women (10% of population) who were selected randomly in conformity to the study criteria. The Cronbach alpha was 0.88.

Vietnamese Menopause Rating Scale (VN-MRS)

Originally, the Menopause Rating Scale (MRS) was developed by the Epidemiology and Health Research Center in Berlin in 1996. It is among the most widely utilized instruments for assessing menopausal symptoms and their impact on quality of life. In 2023, the MRS was adapted and tested for reliability and validity in the Vietnamese language by Hanh and colleagues. The VN-MRS comprises 11 items categorized into three sub-scales: somatic, psychological, and urogenital. The somatic sub-scale encompasses items related to vasomotor symptoms (flushing), cardiac complaints, sleep disturbances, and joint and muscular discomfort (items 1, 2, 3, and 11). The psychological sub-scale includes manifestations such as depressive mood, irritability, anxiety, and both physical and mental exhaustion (items 4, 5, 6, and 7). The urogenital sub-scale addresses sexual dysfunction, bladder complaints, and vaginal dryness (items 8, 9, and 10). Each item is rated on a five-point Likert scale ranging from 0 to 4, where 0 denotes absence or minimal complaints, 1 indicates mild symptoms, 2 reflects moderate severity, 3 signifies severe symptoms, and 4 represents very severe manifestations. The total VN-MRS score is derived by summing the ratings across all 11 items, yielding a possible range from 0 (indicating no symptomatology) to 44 (reflecting extremely severe symptoms). Permission to utilize the VN-MRS was formally obtained from the authors. The VN-MRS Cronbach alpha was 0.89. In our study, the reliability was tested with 40 women, selected randomly in conformity to the study criteria, and the result was 0.87.

### 2.4. Data Collection and Analysis

Data were collected by the primary investigators (PIs) and research assistants (RAs) who were registered nurses who worked at communes/wards. RAs were carefully trained in conducting interviews based on a structured questionnaire to collect data. Participants who met the inclusion criteria were recruited and interviewed face to face to prevent misinterpretation or data loss. Each interview took 15 to 20 min. PIs and RAs maintained neutral vocal and gestural cues to avoid biasing responses; all answers were recorded objectively. Participants were assured that their responses would carry no personal consequences. Data were collected from June 2023 to October 2023. The Statistical Package for Social Sciences version 23 (IBM Corp., Armonk, NY, USA) was used to test the reliability of the instruments, assumption, descriptive statistics, and correlation between the VN-UQOL and VN-MRS. AMOS statistical package version 22 (IBM Corp., Armonk, NY, USA) were used to run CFA for the VN-UQOL.

### 2.5. Ethical Consideration

The study was approved by the Ethics Committee for Biomedical Research of Da Nang University of Medical Technology and Pharmacy, number 01/QD-HDDD. In accordance with ethical research standards, written informed consent was obtained from all participants prior to data collection. Participants were explicitly informed of their unfettered right to decline participation or withdraw from the study at any juncture without consequence. Throughout the entire research process, participant anonymity and the confidentiality of their responses were strictly safeguarded.

## 3. Results

### 3.1. Demographic Characteristics of Participants

The demographic characteristics of the 384 participants are presented in Table 1. The mean age of the sample was 57.77 years (SD = 4.33), with an age range of 46 to 65 years. Regarding educational attainment, the majority of participants (54.43%) had completed secondary school education or lower. In terms of marital status, most participants were married (80.21%). Concerning occupational background, the largest group consisted of homemakers (33.07%). With respect to parity, the vast majority of participants had three or more children (65.89%), while 32.81% had one to two children, and only 1.3% had no children. Analysis of body mass index (BMI) revealed that half of the participants (50.52%) were within the normal weight range, 46.09% were classified as overweight or obese, and 3.39% were underweight. The mean age at menopause was 50.72 years (SD = 3.65). The majority of participants (91.67%) experienced menopause between the ages of 40 and 55 years, while 7.55% experienced menopause after age 55, and a small fraction (0.78%) experienced premature menopause before age 40. The participants was predominantly middle-income (89.8%), with small proportions of poor (2.9%), near-poor (1.8%), and better-off (5.5%) households. Notably, a substantial majority of women (73.4%) reported menstrual irregularities before their final menstrual period. The duration of irregularity was distributed across categories, with 38.7% experiencing 1–6 months, 37.6% experiencing 7–12 months, and 23.8% experiencing more than 12 months.

### 3.2. Reliability

#### 3.2.1. Internal Reliability

The reliability analysis confirms that both the total scale (α = 0.87) and all four sub-scales (α ranging from 0.81 to 0.93) possess strong internal consistency. The item-level statistics support the retention of all 23 items, as each contributes positively to the overall measurement precision (Table 2).

#### 3.2.2. Composite Reliability

Composite reliability values for the four domains ranged from 0.82 to 0.93, all well above the recommended threshold of 0.70, indicating good internal consistency and reliability of the sub-scale scores. The average variance extracted (AVE) exceeded 0.50 for all domains except for the emotional domain (QoL3), where the AVE was 0.47, marginally below the conventional cutoff (Table 3).

### 3.3. Validity

#### 3.3.1. Content Validity

To establish content validity, the synthesized Vietnamese version of the instrument was submitted to a panel of six experts. Specifically, the team included two obstetrician-gynecologists with over 15 years of clinical experience in menopause management and hormone therapy; one psychiatrist specializing in women’s mental health, with extensive work on anxiety and mood disorders in midlife women; one public health researcher with a doctoral degree in psychometrics and scale development who advised on item analysis and content validity indices; and two senior nursing faculty members experienced in the linguistic validation and cultural adaptation of patient-reported outcome measures. All panel members held advanced degrees (MD, PhD, or equivalent) and had previously contributed to instrument development or validation research.

The experts were tasked with evaluating the instructions, response format, and individual items. Content validity was assessed at both the item level (I-CVI) and the scale level (S-CVI). For item-level evaluation, a four-point Likert scale was employed, with ratings defined as follows: 1 = not relevant, 2 = unable to assess relevance, 3 = quite relevant but requiring minor revision, and 4 = highly relevant. Any item receiving a rating of 1 or 2 was identified as requiring revision. The I-CVI for each item was calculated by dividing the number of experts who assigned a rating of either 3 or 4 by the total number of experts (N = 6). The final calculation results of I-CVI and S-CVI were 1.

#### 3.3.2. Construct Validity

Table 4 indicates that all 23 items could be grouped into four factors. Specifically, Factor 1 (VN-UQOL1) comprised seven items, Factor 2 (VN-UQOL2) consisted seven items, Factor 3 (VN-QOL3) included six items, and Factor 4 (VN-QOL4) contained three items. All factor loadings exceeded the minimum recommended threshold of 0.50, indicating that each observed variable significantly contributed to its respective latent construct [8]. The majority of loadings exceeded 0.70.

#### 3.3.3. Discriminant, Known-Group, Theoretical Based, and Convergent Validity

Table 5 indicates that age and age at menopause were not significantly correlated with the UQOL scores, while marked group differences emerged for socioeconomic and health-related variables. Education level, BMI, household economic status, the presence of menstrual irregularities before the final menstrual period, and the duration of those irregularities were all significantly associated with the UQOL scores, with a clear gradient: more disadvantaged socioeconomic positions and longer durations of menstrual irregularity were linked to lower quality of life. Parity showed no significant association.

**Figure 1:**
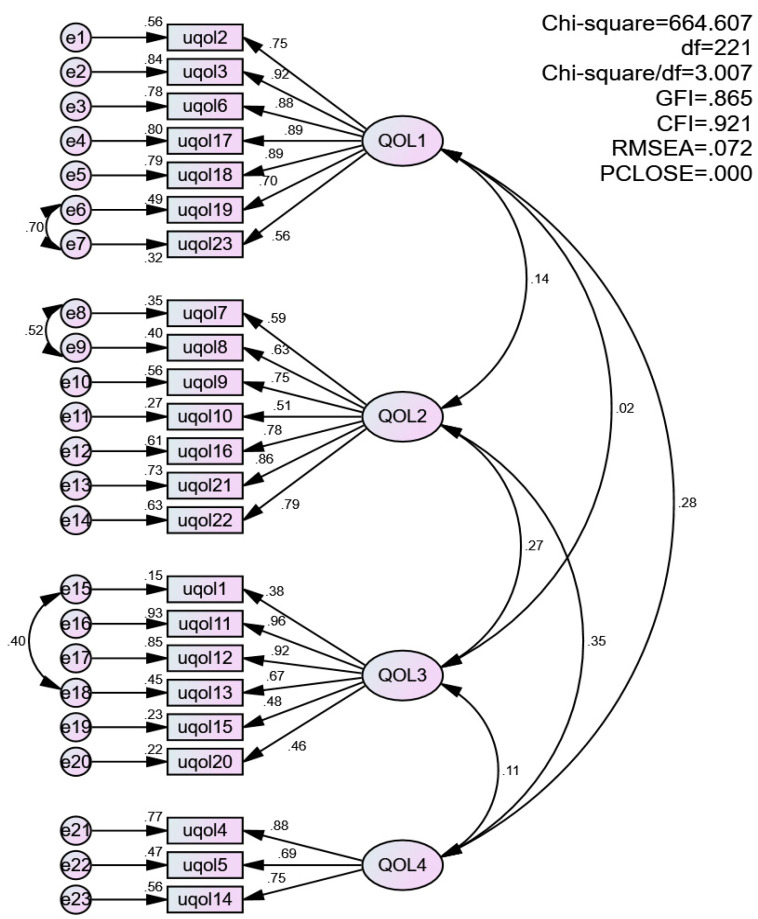
Measurement model and fit indices obtained from confirmatory factor analysis (CFA) of the questionnaire.

Figure 1 presents the model fit indices. The CFA results yielded the following goodness-of-fit indices: χ^2^ = 664.607, df = 221, χ^2^/df = 3.007, GFI = 0.865, CFI = 0.921, and RMSEA = 0.072. These indices collectively indicate that the measurement model achieved an acceptable to good level of fit. The chi-square/degrees of freedom ratio (CMIN/DF) of 3.007 was below the recommended threshold of 5.0, indicating acceptable model fit [6]. The comparative fit index (CFI) of 0.921 surpassed the conventional cutoff of 0.90, demonstrating good incremental fit relative to the null model [6,9]. The root mean square error of approximation (RMSEA) of 0.072 was below the 0.08 threshold for acceptable fit and approached the 0.06 criterion for good fit [6,9]. The goodness of fit index (GFI) of 0.865 slightly exceeded the 0.80 acceptable threshold but fell below the ideal 0.90 criterion.

Table 6 presents the results of the CFA model comparison. One-factor model did not fit the data, as reflected by its poor fit indices. In contrast, the correlated four-factor model demonstrated an acceptable level of fit and was substantially superior to the one-factor model. The higher-order model also achieved an acceptable fit, with indices nearly equivalent to those of the four-factor model.

### 3.4. Concurrent Validity

The concurrent validity of the VN-UQOL was assessed by examining correlations between its overall and sub-scale scores and those of the VN-MRS. Specifically, the VN-UQOL emotional sub-scale was correlated with the VN-MRS psychological domain, the VN-UQOL health sub-scale with the VN-MRS somato-vegetative domain, the VN-UQOL sexual sub-scale with the VN-MRS urogenital domain, and the overall VN-UQOL score with the overall VN-MRS score. To establish concurrent validity, statistically significant negative correlations were hypothesized for each of these paired comparisons. The resulting correlation matrix is presented in Table 7.

## 4. Discussion

The present study aimed to translate, culturally adapt, and psychometrically validate the Vietnamese version of the Utian Quality of Life (VN-UQOL) Scale among community-dwelling postmenopausal women in Da Nang, Vietnam. The findings demonstrate that the VN-UQOL possesses robust psychometric properties, with strong internal consistency and satisfactory construct and concurrent validity, supporting its utility as a culturally appropriate instrument for assessing menopause-specific quality of life in Vietnamese women.

### 4.1. Reliability of the VN-UQOL

The total VN-UQOL demonstrated good internal consistency (Cronbach’s α = 0.87), exceeding the 0.70 threshold [5] and comparable to values reported for the original (α = 0.83) [1], Turkish (α = 0.86) [2], and Yoruba (α = 0.88) versions [3]. Examination of the four sub-scales revealed Cronbach’s alpha values ranging from 0.81 to 0.93, all demonstrating acceptable to good internal consistency. The occupational quality of life sub-scale showed exceptional internal consistency (α = 0.93), substantially exceeding the values reported in the original UQOL validation (α = 0.81) [1] and the Turkish adaptation (α = 0.85) [2]. This notably high reliability is particularly meaningful when considered within the Vietnamese sociocultural context, where middle-aged women frequently navigate multiple concurrent roles as family caregivers, household income contributors, and participants in both formal and informal economic sectors. The occupational composition of the present sample—comprising around 33% homemakers and 19.0% vendors, may reflect this role multiplicity and suggests that the items pertaining to work fulfillment, societal contribution, and personal recognition held particular clarity and relevance for these respondents. In Vietnam’s rapidly developing economy, which has drawn increasing numbers of women into the workforce, occupational identity increasingly intersects with family responsibilities and social status, potentially rendering this domain especially coherent and meaningful for menopausal women.

The health-related quality of life sub-scale demonstrated good reliability (α = 0.88), consistent with previous validations. This sub-scale encompasses items addressing physical well-being, diet, exercise, and perceived control over health—domains that may be especially relevant in the Vietnamese context, where traditional health beliefs emphasizing balance and self-care coexist with increasing exposure to biomedical models of health [10]. Demographic data also revealed that most of participants in the study reported BMI in normal values with 50.52%. This demonstrates consistency in the collected data. The emotional quality of life sub-scale showed satisfactory internal consistency (α = 0.84), slightly lower than the occupational and health sub-scales but well within acceptable limits. This pattern mirrors findings from the Yoruba adaptation, where the emotional sub-scale also demonstrated relatively lower reliability (α = 0.75) [3], suggesting that affective domains may be more susceptible to cultural variation in expression and interpretation.

The sexual quality of life sub-scale, comprising four items, demonstrated acceptable reliability (α = 0.81). Although this represented the lowest alpha among the four sub-scales, it nonetheless exceeded the 0.80 criterion for good reliability and compared favorably with the original UQOL (α = 0.66) [1] and the Turkish version (α = 0.76) [2]. The improved reliability in the Vietnamese context may reflect careful attention to culturally sensitive wording during the translation process, particularly for items addressing sexual satisfaction and discomfort. However, it is noteworthy that item 15 (“I currently experience physical discomfort or pain during sexual activity”) exhibited the lowest corrected item-total correlation (0.30) and would marginally increase the overall alpha if deleted (0.88). This finding resonates with observations from the Yoruba adaptation, where the sexual sub-scale showed weak convergent validity, attributed to cultural norms surrounding the discussion of sexual matters [3]. In Vietnamese culture, where traditional values emphasize modesty and reserve in discussing intimate topics, some women may have experienced discomfort responding to the sexual items, potentially introducing measurement noise. Nevertheless, the decision to retain all items was justified by the negligible improvement upon deletion and the theoretical importance of sexual well-being and emotion quality of life related to sexual activities as a component of menopause-specific quality of life.

Item-level analysis revealed corrected item-total correlations ranging from 0.30 to 0.60, with the majority exceeding the minimum acceptable threshold of 0.30 [6]. Item 21 (“I feel physically well”) demonstrated the highest correlation (0.60), underscoring the centrality of perceived physical well-being to overall quality of life in this population. Items 3, 5, 16, 17, 18, 21, and 22 all exhibited correlations exceeding 0.50, indicating strong contributions to the overall scale. The narrow range of Cronbach’s alpha if the item is deleted (0.86 to 0.88) confirms that no single item disproportionately influences the scale’s reliability, supporting the retention of all 23 items and affirming the scale’s internal coherence.

To complement the conventional Cronbach’s alpha estimates, composite reliability (CR) and average variance extracted (AVE) were performed. All four domains demonstrated strong composite reliability (CR = 0.818–0.928), confirming that the sub-scale scores are reliable. AVE values supported convergent validity for the occupational, health, and sexual domains (AVE ≥ 0.506), while the emotional domain exhibited an AVE of 0.469, slightly below the 0.50 benchmark. This marginally lower AVE suggests that the emotional items capture a somewhat broader range of affective symptoms, which may dilute shared variance among them. Nevertheless, the CR for this domain remained satisfactory, indicating that the domain is still coherent. Combined with discriminant validity evidence, these findings paint a more complete psychometric picture and confirm that the Vietnamese UQOL possesses adequate convergent and discriminant properties, beyond what internal consistency alone can convey.

### 4.2. Validity of the VN-UQOL

The content validity of the VN-UQOL was rigorously established through expert panel review, yielding perfect item-level content validity index (I-CVI) and scale-level content validity index (S-CVI) values of 1.0. These results substantially exceeded the minimum acceptable thresholds of 0.78 for I-CVI and 0.90 for S-CVI [11], indicating unanimous expert agreement on the relevance and appropriateness of all items, instructions, and response formats. The panel’s composition—six Vietnamese experts with specialized knowledge in menopause, women’s health—and psychometrics ensured comprehensive evaluation from both clinical and methodological perspectives. This strong content validity provides an essential foundation for subsequent psychometric evaluation, confirming that the instrument adequately represents the full domain of menopause-specific quality of life as conceptualized in the Vietnamese cultural context.

The pattern of associations observed in this analysis offers multifaceted validity evidence for the Vietnamese UQOL. Importantly, the absence of significant correlations with chronological age and age at menopause serves as evidence of discriminant validity, demonstrating that the UQOL is not simply a proxy for these demographic variables. This aligns with the scale’s conceptual foundation, which positions menopause-specific quality of life as a distinct construct rooted in symptom experience and health perception rather than in age itself [1].

In contrast, the significant differences detected across socioeconomic and health-related groups furnish robust known-group validity. Women with education at the secondary school level or below reported significantly lower scores than their more highly educated counterparts, and a clear economic gradient emerged, the better-off group scored markedly higher than poor and near-poor women. These findings are highly consistent with the broader health-inequality literature in Vietnam, where lower educational attainment and constrained financial resources limit access to health information, preventive services, and social support, all of which negatively affect postmenopausal well-being [12]. Furthermore, BMI category was significantly associated with the UQOL scores (*p* < 0.001). Women classified as overweight or obese reported the lowest mean UQOL score, significantly lower than those with normal weight and underweight. This is consistent with the broader literature. The elevated body mass may contribute to a higher burden of vasomotor symptoms, joint pain, cardiovascular risk, and psychological distress, all of which can reduce perceived well-being during the menopausal transition [13]. The UQOL’s sensitivity to such socioeconomic gradients and health parameters confirms its ability to distinguish between groups known to differ in health-related quality of life.

The strongest support for known-group and theoretically based validity came from variables tied to the menopausal transition itself. The presence of menstrual irregularities before the final menstrual period was associated with a significantly lower UQOL score, and a dose–response relationship with the duration of irregularity was evident: the longer the irregularity persisted, the poorer the quality of life. This mirrors prospective evidence that prolonged menstrual irregularity reflects a more symptomatic perimenopausal phase with lasting negative effects on well-being [14]. In the Vietnamese socio-cultural context, menstrual disruptions may carry additional psychological burden due to anxiety and stigma around reproductive health changes, which could amplify their impact on daily functioning. The ability of the Vietnamese UQOL to detect these graded associations not only reproduces findings from international UQOL research, but also extends them by confirming a duration-dependent relationship. Taken together, these results demonstrate that the adapted instrument relates to theoretically relevant external variables in the expected manner, thereby providing a compelling body of evidence for its convergent, discriminant, and known-group validity within the construct validation framework.

Confirmatory factor analysis (CFA) provided evidence for the construct validity of the VN-UQOL, supporting the hypothesized four-factor structure originally proposed by Utian and colleagues (2018). All 23 items loaded significantly on their respective factors, with standardized factor loadings ranging from 0.56 to 0.92, exceeding the minimum recommended threshold of 0.50 [6]. The majority of loadings surpassed 0.70, demonstrating acceptable convergent validity across all four domains. Particularly robust loadings were observed for items measuring occupational quality of life (items 3, 6, 17, and 18; loadings 0.89–0.91) and emotional quality of life (items 11 and 13; loadings 0.92 and 0.81), indicating that these items are especially powerful indicators of their respective constructs.

The factor structure observed in this Vietnamese sample closely parallels those reported in previous validations. The occupational factor (VN-UQOL1) comprised seven items (2, 3, 6, 17, 18, 19, 23) with loadings from 0.70 to 0.91, consistent with the original UQOL factor structure [1] and the Turkish adaptation [2]. The health factor (VN-UQOL2) included seven items (7, 8, 9, 10, 16, 21, 22) with loadings from 0.58 to 0.84, demonstrating that items addressing diverse aspects of physical well-being, nutrition, exercise, and perceived health control coalesce into a coherent domain. The emotional factor (VN-UQOL3) comprised six items (1, 11, 12, 13, 15, 20) with loadings from 0.56 to 0.92, confirming that mood states, anxiety, perceived control, and expectations for the future constitute a unified affective dimension. The sexual factor (VN-UQOL4) included three items (4, 5, 14) with loadings from 0.69 to 0.92, replicating the structure of the original UQOL and supporting the conceptualization of sexual well-being as a distinct domain of menopause-specific quality of life.

Model fit indices indicated that the four-factor measurement model achieved acceptable to good fit across multiple criteria. The chi-square/degrees of freedom ratio (CMIN/DF = 3.007) fell below the recommended threshold of 5.0, indicating acceptable model fit [6]. The comparative fit index (CFI = 0.921) surpassed the conventional cutoff of 0.90, demonstrating good incremental fit relative to the null model [9]. The root mean square error of approximation (RMSEA = 0.072) was below the 0.08 threshold for acceptable fit and approached the 0.06 criterion for good fit [6,9]. The goodness of fit index (GFI = 0.865) slightly exceeded the acceptable threshold of 0.80, though it fell short of the ideal 0.90 criterion. This pattern of fit indices is comparable to those reported in the Turkish validation (CFI = 0.91, RMSEA = 0.07) [2] and the Yoruba validation (CFI = 0.89, RMSEA = 0.08) [3], suggesting that the four-factor structure of the UQOL is replicable across diverse cultural contexts, albeit with minor variations attributable to sample characteristics and cultural factors.

The marginally lower GFI observed in this study may reflect the complexity of the measurement model relative to sample size, or may indicate areas where cultural adaptation could be further refined. However, given that multiple fit indices converged in support of adequate model fit, and that all factor loadings were statistically significant and theoretically meaningful, the CFA results provide strong evidence for the construct validity of the VN-UQOL.

Furthermore, we tested a set of competing models including a unidimensional one-factor model, the original four-factor model, and a higher-order model in which the four first-order factors load onto a general quality-of-life factor. The one-factor model demonstrated poor fit across all indices (χ^2^/df = 11.135, CFI = 0.599, RMSEA = 0.163), confirming that menopausal quality of life as measured by the UQOL cannot be reduced to a single homogeneous dimension. In contrast, the four-factor model showed acceptable fit, supporting the original four-domain structure. Notably, the higher-order model yielded nearly equivalent fit indices (χ^2^/df = 3.040, CFI = 0.919, RMSEA = 0.073), indicating that the four domain-specific factors can be adequately explained by a general overarching quality-of-life factor. This pattern is consistent with the theoretical premise that, while menopausal quality of life encompasses distinct facets (occupational, health, emotional, and sexual), these domains are interrelated and contribute to an integrated, global perception of well-being. The near-equivalence of the four-factor and higher-order models suggests that both representations are tenable; the higher-order structure offers a parsimonious account of the substantial correlations among the sub-scales. These findings deepen the construct validity evidence for the Vietnamese UQOL by demonstrating that the instrument captures both a multifaceted and coherent latent quality-of-life construct.

Concurrent validity was established through examination of the Pearson correlations between the VN-UQOL and the Vietnamese Menopause Rating Scale (VN-MRS), a previously validated instrument assessing menopausal symptom severity [15]. As hypothesized, all correlations were statistically significant and negative, indicating that higher quality of life was associated with lower symptom burden. The overall VN-UQOL score correlated moderately with the overall VN-MRS score (r = −0.40, *p* < 0.001), demonstrating that the two instruments capture related but distinct constructs, quality of life and symptom severity, as theoretically expected.

Domain-specific correlations followed the hypothesized pattern, providing evidence for convergent and discriminant validity. The VN-UQOL health sub-scale correlated significantly with the VN-MRS somato-vegetative domain (r = −0.36, *p* < 0.001), confirming that health-related quality of life is inversely related to somatic and vasomotor symptoms. The VN-UQOL emotional sub-scale correlated with the VN-MRS psychological domain (r = −0.16, *p* < 0.01), though this correlation was weaker than anticipated. This modest association may reflect cultural factors in emotional expression and symptom reporting among Vietnamese women. Research on menopause experiences in Vietnam suggests that psychological symptoms may be somatized or attributed to life circumstances rather than explicitly recognized as menopause-related [10,16]. Consequently, the relationship between self-reported emotional quality of life and psychological symptom severity may be attenuated by cultural patterns of symptom interpretation and communication.

The VN-UQOL sexual sub-scale correlated significantly with the VN-MRS urogenital domain (r = −0.19, *p* < 0.001), confirming that sexual quality of life is inversely related to urogenital symptoms. The modest magnitude of this correlation, while statistically significant, warrants consideration. In the original UQOL validation, the sexual sub-scale also demonstrated relatively weaker correlations with symptom measures, attributed in part to the small number of items [1]. Additionally, cultural factors likely play a role. In Vietnamese society, where discussions of sexuality remain largely private and conservative norms prevail, women may underreport sexual concerns or may not readily connect urogenital symptoms with sexual quality of life [16,17]. This interpretation aligns with findings from the Yoruba adaptation, where the sexual sub-scale similarly showed weak convergent validity, leading the authors to suggest that “culture-bound norms around discussing sexual life” may constrain measurement precision in this domain [3].

The pattern of correlations observed in this study show that significant negative associations of modest magnitude are consistent with previous cross-cultural validations of the UQOL. The Turkish validation reported correlations ranging from −0.21 to −0.45 between the UQOL sub-scales and symptom measures [2], while the Yoruba validation found correlations from −0.18 to −0.38 [3]. These consistent findings across diverse populations suggest that menopause-specific quality of life, as measured by the UQOL, is related to but distinct from menopausal symptom severity, supporting the instrument’s unique contribution to a comprehensive assessment of menopausal well-being.

The demographic characteristics of the study sample merit consideration in interpreting these findings. Participants had a mean age of 57.8 years, with the majority married (80.2%), homemakers (33.1%), and having three or more children (65.9%). These characteristics reflect broader demographic patterns among Vietnamese women of menopausal age, among whom high parity, traditional family roles, and transitions from agricultural or informal sector work to retirement or homemaking are common [10]. The predominance of homemakers and vendors in the sample underscores the importance of the occupational quality of life sub-scale, which in this context captures not only formal employment, but also satisfaction with daily activities, sense of purpose, and recognition within family and community networks.

The mean age at menopause in this sample (50.7 years) closely approximates estimates from other Vietnamese studies [17,18,19] and falls within the typical range for Asian populations (49–51 years). The high prevalence of normal BMI (50.52%) is consistent with findings of the health sub-scale items addressing physical well-being, diet, and exercise, which capture dimensions of health-related quality of life particularly salient in a population facing rising metabolic risk.

In summary, the VN-UQOL Scale demonstrates strong psychometric properties, with good internal consistency, robust content validity, satisfactory construct validity confirmed through confirmatory factor analysis, and acceptable concurrent validity with a menopausal symptom measure. The instrument retains the four-factor structure of the original UQOL while achieving cultural appropriateness through rigorous translation and adaptation processes. These findings support the VN-UQOL as a reliable and valid instrument for assessing menopause-specific quality of life in Vietnamese women, addressing a critical methodological gap and providing a valuable tool for clinical practice and research in Vietnam.

## 5. Limitations and Future Research

There are several limitations in this study. First, the cross-sectional design precluded an assessment of test–retest reliability and sensitivity to change, and all analyses were conducted on a single dataset, which may have slightly inflated the model fit indices. Relatedly, although the four-factor model demonstrated acceptable fit, this was achieved in part by allowing correlations between selected error terms. Such adjustments suggest possible item redundancy, and the stability of the factor structure would benefit from confirmation in an independent sample. Second, the sample was drawn exclusively from one urban center (Da Nang) and excluded women with a history of hormone therapy or diagnosed psychiatric disorders. While these criteria reduced confounding in this initial validation, they may limit generalizability to the broader Vietnamese postmenopausal population. Future research should employ longitudinal, multi-site designs with heterogeneous samples, include test–retest and responsiveness assessments, cross-validate the model, and add more variables regarding health parameters such as lifestyle, habits, therapies, comorbidities, or hormonal status to further substantiate the scale’s psychometric robustness and generalizability.

## 6. Conclusions

This study provides initial evidence supporting the reliability and construct validity of the Vietnamese Utian Quality of Life Scale (VN-UQOL) in postmenopausal women. The results indicate that the VN-UQOL is a promising culturally adapted instrument for assessing menopause-specific quality of life across the occupational, health, emotional, and sexual domains. Given its brevity and simple Likert-scale format, the scale can readily be adopted by clinicians to identify women whose perceived well-being is substantially compromised and to monitor individual responses to interventions. Notably, the Vietnamese government has recently issued a policy mandating annual general health check-ups for all citizens starting from 2026. In this context, the VN-UQOL—being short, self-administered, and easy to score—could serve as a practical screening tool to detect menopause-related quality-of-life impairments at the population level during routine check-ups, thereby facilitating early referral and timely supportive care within the primary healthcare system.

## Figures and Tables

**Table 1:** Demographic characteristics of the participants (*n* = 384).

Characteristics		Frequency	Percentage
Age Mean ± SD (Min–Max): 57.770 ± 4.330 (46–65)			
Educational level	Secondary school education or lower	209	54.43
	High school education or higher	175	45.57
Marital status	Married	308	80.21
	Widowed	46	11.98
	Divorced	25	6.51
	Single	5	1.30
Occupational status	Famers	50	13.02
	Vendors	73	19.01
	Homemakers	127	33.07
	Maritime	1	0.26
	Officers	40	10.42
	Retirees	41	10.68
	Others	52	13.54
Parity	No child	5	1.30
	1–2 children	126	32.81
	Three or more children	253	65.89
BMI	Underweight	13	3.39
	Normal	194	50.52
	Overweight or obese	177	46.09
Age of menopause Mean ± SD: 50.72 ± 3.65 (year)			
Age group of menopause	<40 age	3	0.78
	40–55 age	352	91.67
	>55 age	29	7.55
Household economic status	Poor	11	2.9
	Near poor	7	1.8
	Middle-income	345	89.8
	Better-off	21	5.5
Presence of menstrual irregularities before the final menstrual period	Yes	282	73.4
	No	102	26.6
Number of months of menstrual irregularity prior to the complete cessation of menstruation	1–6 months	109	38.7
	7–12 months	106	37.6
	>12 months	67	23.8

**Table 2:** Internal reliability of the VN-UQOL.

Item	Sub-Scale	Correlated Item-Total Correlation	Cronbach’s Alpha if Item Deleted
1. I am able to control things in my life that are important to me.	3	0.39	0.86
2. I feel challenged by my work.	1	0.46	0.86
3. I believe my work benefits society.	1	0.54	0.86
4. I am not content with my sexual life.	4	0.41	0.86
5. I am content with my romantic life.	4	0.53	0.86
6. I have gotten a lot of personal recognition in my community or at my job.	1	0.49	0.86
7. I am unhappy with my appearance.	2	0.39	0.86
8. My diet is not nutritionally sound.	2	0.44	0.86
9. I feel in control of my eating behavior.	2	0.49	0.86
10. Routinely, I engage in active exercise 3 or more times each week.	2	0.35	0.87
11. My mood is generally depressed.	3	0.34	0.87
12. I frequently experience anxiety.	3	0.33	0.87
13. Most things that happen to me are out of my control	3	0.40	0.86
14. I am content with the frequency of my sexual interactions with a partner.	4	0.36	0.87
15. I currently experience physical discomfort or pain during sexual activity.	3	0.3	0.88
16. I believe I have no control over my physical health.	2	0.52	0.86
17. I am proud of my occupational accomplishments.	1	0.52	0.86
18. I consider my life stimulating.	1	0.53	0.86
19. I continue to set new personal goals for myself.	1	0.46	0.86
20. I expect that good things will happen in my life.	3	0.42	0.86
21. I feel physically well.	2	0.60	0.86
22. I feel physically fit.	2	0.54	0.86
23. I continue to set new professional goals for myself.	3	0.34	0.87
Cronbach alpha for scale: 0.87 Cronbach alpha for sub-scale “Occupational quality of life” (sub-scale 1): 0.93 Cronbach alpha for sub-scale “Health quality of life” (sub-scale 2): 0.88 Cronbach alpha for sub-scale “Emotional quality of life” (sub-scale 3): 0.84 Cronbach alpha for sub-scale “Sexual quality of life” (sub-scale 4): 0.81			

**Table 3:** Composite reliability and average variance extracted.

Factor	Number of Item	CR	AVE	Evaluation
QoL1	7	0.93	0.65	Good
QoL2	7	0.88	0.51	Acceptable
QoL3	6	0.83	0.47	Acceptable CR, Low AVE
QoL4	3	0.82	0.60	Good

**Table 4:** Factor loading for the VN-UQOL.

Item	VN-UQOL 1	VN-UQOL 2	VN-UQOL 3	VN-UQOL 4
1. I am able to control things in my life that are important to me.			0.56	
2. I feel challenged by my work.	0.79			
3. I believe my work benefits society.	0.90			
4. I am not content with my sexual life.				0.92
5. I am content with my romantic life.				0.69
6. I have gotten a lot of personal recognition in my community or at my job.	0.91			
7. I am unhappy with my appearance.		0.76		
8. My diet is not nutritionally sound.		0.82		
9. I feel in control of my eating behavior.		0.84		
10. Routinely, I engage in active exercise 3 or more times each week.		0.58		
11. My mood is generally depressed.			0.92	
12. I frequently experience anxiety.			0.69	
13. Most things that happen to me are out of my control			0.81	
14. I am content with the frequency of my sexual interactions with a partner.				0.89
15. I currently experience physical discomfort or pain during sexual activity.			0.65	
16. I believe I have no control over my physical health.		0.77		
17. I am proud of my occupational accomplishments.	0.89			
18. I consider my life stimulating.	0.91			
19. I continue to set new personal goals for myself.	0.78			
20. I expect that good things will happen in my life.			0.59	
21. I feel physically well.		0.81		
22. I feel physically fit.		0.75		
23. I continue to set new professional goals for myself.	0.70			

**Table 5:** Correlations between the participants’ characteristics and UQL score (*n* = 384).

Factor		r	Mean ± SD	*p*
Age		−0.07		0.167 *
Age of menopause		0.017		0.74 *
Educational level	Secondary school education or lower		90.01 ± 10.3	0.003 **
	High school education or higher		93.18 ± 10.3	
Parity	No child		93.00 ± 14.6	0.355 ***
	1–2 children		92.5 ± 11.0	
	Three or more children		90.91 ± 9.9	
BMI	Underweight		92.54 ± 13.5	
	Normal		94.32 ± 10.1	*p* < 0.001 ***
	Overweight or obese		177 ± 9.5	
Household economic status	Poor		86.8 ± 8.9	1
	Near poor		86.29 ± 9.4	1
	Middle-income		91.31 ± 10.4	0.48
	Better-off		98.0 ± 8.0	0.01 ***
Presence of menstrual irregularities before the final menstrual period	Yes		90.18 ± 10.4	*p* < 0.001 **
	No		95.0 ± 9.6	
Number of months of menstrual irregularity prior to the complete cessation of menstruation	1–6 months		93.65 ± 9.6	1
	7–12 months		89.64 ± 10.4	0.009 ***
	>12 months		85.37 ± 9.7	*p* < 0.001 ***

* Spearman Rho; ** *t*-test; *** ANOVA (post hoc).

**Table 6:** Comparison of the CFA model.

Model	χ^2^/df	GFI	CFI	TLI	RMSEA	Evaluation
One factor	11.135	0.548	0.599	00.54	0.163	Poor
Four factors	3.007	0.865	0.921	0.910	0.072	Acceptable
Higher-order	3.040	0.864	0.919	0.908	0.073	Acceptable, nearly equivalent to the four-factor model

**Table 7:** Pearson correlation of the VN-UQOL and VN-MRS.

VN-UQOL	VN-MRS			
	Somato-Vegetative Domain	Psychological Domain	Urogenital Domain	Overall
Health sub-scale	−0.36 ***			
Emotional sub-scale		−0.16 **		
Sexual sub-scale			−0.19 ***	
Overall				−0.40 ***

** *p* < 0.01; *** *p* < 0.001.

## Data Availability

The data presented in this study are available on request from the first author due to institutional data ownership and participant confidentiality.
